# A Primer on Network Meta-Analysis for Dental Research

**DOI:** 10.5402/2012/276520

**Published:** 2012-06-21

**Authors:** Yu-Kang Tu, Clovis Mariano Faggion

**Affiliations:** ^1^Department of Oral Biology, Leeds Dental Institute, Leeds LS2 9JT, UK; ^2^Division of Biostatistics, Leeds Institute of Genetics, Health and Therapeutics, University of Leeds, Leeds LS2 9JT, UK; ^3^Department of Oral Sciences, Faculty of Dentistry, University of Otago, P.O. Box 647, Dunedin 9054, New Zealand

## Abstract

In the last decade, a new statistical methodology, namely, network meta-analysis, has been developed to address limitations in traditional pairwise meta-analysis. Network meta-analysis incorporates all available evidence into a general statistical framework for comparisons of all available treatments. A further development in the network meta-analysis is to use a Bayesian statistical approach, which provides a more flexible modelling framework to take into account heterogeneity in the evidence and complexity in the data structure. The aim of this paper is therefore to provide a nontechnical introduction to network meta-analysis for dental research community and raise the awareness of it. An example was used to demonstrate how to conduct a network meta-analysis and the differences between it and traditional meta-analysis. The statistical theory behind network meta-analysis is nevertheless complex, so we strongly encourage close collaboration between dental researchers and experienced statisticians when planning and conducting a network meta-analysis. The use of more sophisticated statistical approaches such as network meta-analysis will improve the efficiency in comparing the effectiveness between multiple treatments across a set of trials.

## 1. Introduction

With the rise of evidence-based medicine movement in the last two decades, systematic reviews and meta-analyses have been widely used for synthesis of evidence on beneficial and/or harmful effects of different treatments. Results from those reviews and meta-analyses provide important information for drawing clinical guidelines and making health policy recommendations. For most clinical conditions, several interventions (which may be drugs, medical devices, surgeries, or a combination of them) are usually available, but most systematic reviews of randomised controlled trials (RCTs) tend to limit their scopes by only evaluating two active treatments or comparing one treatment to a control. Even if a systematic review evaluates multiple treatments, traditional meta-analysis can only perform pairwise comparisons.

There are several limitations to this approach [1–4]. For instance, suppose there are three new and more expensive treatments A, B, and C and a standard treatment D, six pair-wise metaanalyses (A-B, B-C, A–C, A–D, B–D, and C-D) may be undertaken to compare the differences for pairs of the four treatments. None or few of included RCTs in the paper would have compared all four treatments, and most RCTs compared only 2 or 3 of them. Consequently, those pairwise meta-analyses use different sets of RCTs for each comparison, and the evidence base is therefore different across all comparisons. A possible consequence is that results from multiple pairwise meta-analyses may not be consistent: for example, in three pairwise comparisons, treatment A is shown to be better than treatment B, and B better than treatment C; but A is inferior to C. Secondly, some head-to-head trials may not have been conducted yet (especially between the new treatments), so it is not possible to undertake traditional pairwise meta-analysis for these comparisons. Thirdly, because the number of studies available for pairwise comparisons is few, each meta-analysis may not have sufficient power to detect any genuine difference between treatments, yielding inconclusive results and providing no useful guidance on decision making.

In the last decade, a new statistical methodology, namely, network meta-analysis, has been developed to address those limitations [5–7]. Network meta-analysis incorporates all available evidence into a general statistical framework for comparisons of all available treatments. Thus, network meta-analysis may play an important role in the improvement of the decision making process by optimizing the use of the existing data. A further development in the network meta-analysis is to use a Bayesian statistical approach, which provides a more flexible modelling framework to take into account of heterogeneity in the evidence and complexity in the data structure [1–4].

Although systematic reviews with network meta-analysis for evidence synthesis has been published in mainstream medical journals [8–12], many dental researchers are still not aware of this new methodology, and, to the best of our knowledge, only a few network meta-analyses have appeared in dental journals [13–16]. The aim of this paper is therefore to provide a nontechnical introduction to network meta-analysis for dental research community and raise the awareness of it. In the next sections, we first explained the rationale and assumptions behind the network meta-analysis; then, we described the statistical model for the network meta-analysis and used an example from periodontology for illustration. In the final section, we discussed a few practical issues to be considered when conducting a network meta-analysis.

## 2. Network Meta-Analysis

**Figure 1:**
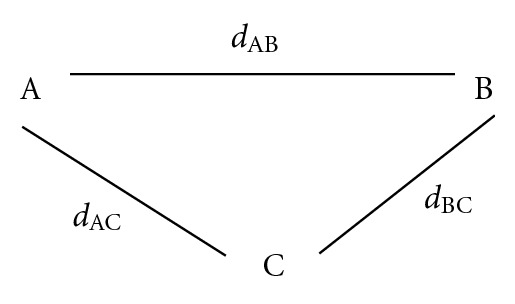
Diagram for the network of three treatments A, B, and C. *d*_AB_, *d*_BC_, and *d*_AC⁡_ are the differences in treatment effect between A and B, between B and C, and between A and C, respectively.

The basic rationale behind network meta-analysis is simple: suppose we have three treatments A, B, and C. Results from RCTs comparing A and B provide direct evidence on the difference in the treatment effects between A and B. In contrast, results from RCTs comparing A–C and those comparing B-C provide indirect evidence on the difference between A and B. The three treatments A, B, and C therefore form a network for treatment effect comparisons (Figure 1). Let us use *d*_AB_, *d*_AC⁡_, and *d*_BC_ to denote the differences in the treatment effects for A-B, A–C, and B-C comparisons, respectively. The difference in treatment effects between A and B from *indirect *comparisons is therefore *d*_AC⁡_ − *d*_BC_ = *d*_AB_^Ind^. If *d*_AB_ is similar to *d*_AB_^Ind^, the direct and indirect evidence is consistent; otherwise, there is *inconsistency* in the evidence, that is, results from direct and indirect evidence are not the same.

### 2.1. Assumptions behind the Network Meta-Analysis

There are several assumptions for the network meta-analysis to yield meaningful results [1–4]. The first assumption is *homogeneity *for standard meta-analysis, that is, all A–C trials are “comparable” and all B-C trials are “comparable.” This is a universal assumption for all meta-analysis, although some heterogeneity increases the generalizability of results. However, whilst evidence may be homogeneous within a set of trials for certain pairwise comparisons, but it may not be so across sets of trials within the network. This leads us to the *similarity* assumption for the network meta-analysis, that is, the included trials are clinically and methodologically similar in term of key factors that modify the response to a treatment, such as patients' characteristics, study settings, lengths of followup, and outcome measurements. In other words, potential confounders for treatment effect comparisons are similarly distributed across included studies. When these two assumptions are questionable, results from direct and indirect evidence may be inconsistent, and consistency is the third assumption that will be discussed in the next section.

It has been suggested that results from the network meta-analysis may be less trustworthy than results from multiple pairwise comparisons, because indirect evidence is less reliable than direct evidence and more prone to biases [17, 18]. Whilst we agree that interpretation of results from the network meta-analysis always needs to be cautious because of potential biases, network meta-analysis is no more prone to biases that traditional meta-analysis, for instance, heterogeneity is also common in traditional meta-analysis. This is because the distinction between direct and indirect evidence is not meaningful in multiple treatments comparisons, as direct evidence for one comparison becomes indirect for some other comparisons [1, 2]. For example, trials including A and C are direct evidence for A–C comparisons, and trials including A and B are evidence for A-B comparisons, but they are also indirect evidence for B-C comparison. So the argument that results from mixed treatment comparison are less reliable than those from direct comparisons is untenable. We do agree that because the scope of a network meta-analysis is much broader than a traditional pairwise meta-analysis and a greater number of studies is included in the analysis, more resources and efforts are required to minimize potential errors and biases in the literature search, quality assessment, data analysis, and final interpretation of results [19, 20].

### 2.2. Inconsistency between Direct and Indirect Evidence

Consistency in direct and indirect evidence is another assumption behind network meta-analysis. Suppose results from trials comparing A with B show A is on average better than B, and trials comparing B with C show B is on average better than C, and indirect comparisons will then show A is better than C. If trials comparing A with C also show A is on average better than C, the indirect and direct evidence is consistent. However, what if direct evidence shows C is better than A? Does this contradiction mean that evidence from indirect comparisons is unreliable and should be disregarded?

If this argument is true, it implies the direct comparisons for A-B and B-C cannot be trusted either. This can be applied to all pairwise comparisons in the network. Therefore, when there is inconsistency in the direct and indirect evidence, the issue is not whether indirect comparisons are less reliable but how this inconsistency may be explained. If trials involved in the indirect comparisons have a better quality and fewer biases, results from indirect evidence may be more reliable than those from direct evidence. Heterogeneity in study protocols (e.g., doses, follow-up time, etc.), patient populations (age, underlying medical conditions, clinical settings, etc.), and methods for the assessment of outcomes is likely to be the source for inconsistency, while random variations may also cause inconsistency. Therefore, it is imperative to check consistency in evidence when undertaking network meta-analysis, and we will explain later in this paper how to conduct a simple test for checking it. Interpretation of network meta-analysis should always take inconsistency into account as the interpretation of traditional meta-analysis should take heterogeneity into account [21–23]. Assessment of quality and characteristics of included trials should be undertaken for network meta-analysis; thus, heterogeneity in evidence can be explored when inconsistency occurs.

### 2.3. Statistical Model for Network Meta-Analysis

Different statistical approaches have been proposed in the literature for comparing multiple treatments [5, 7]. A fixed effects model assumes a common effect behind the observed effects, while a random effects model assumes that the “true” effect follows a distribution. In this section, we introduce the random effects model approach as suggested by Whitehead [24]:

(1) $$\begin{array}{c} f\left(y_{ijk}\right)=u+s_{i}+t_{j}+{\left(st\right)}_{ij},\; \end{array}$$

where *y*_*ijk*_ is the outcome for subject *k* in treatment group *j* in study *i*, *u* the grand mean, *s*_*i*_ the study effect, *t*_*j*_ treatment effect, and *st*_*ij*_ interaction between study *i* and treatment *j*. The function *f* defines the relation between the outcome *y* and effects of *s*, *t*, and *st*. In meta-analysis, individual patient data are usually not available, and summary statistics for treatment effect, such as means or odds, are used instead. Therefore, (1) can be rewritten as

(2) $$\begin{array}{c} \theta_{ij}=u+s_{i}+t_{j}+{\left(st\right)}_{ij},\; \end{array}$$

where *θ*_*ij*_ is the treatment effects for treatment *j* in the study *i*. For example, *θ*_*ij*_ may be mean probing pocket depth reduction, or log odds for dental implant failure in 3-year followup. The interpretation of (2) is that the average treatment effect for all treatments is *u*, and for a specific treatment arm *j* in one study, its treatment effects depends on the study where it is applied (i.e., study characteristics affect the performance of all treatments in that specific study), treatment (treatments have different genuine effects), and the interaction between study and treatment (the effect of treatment *j* varies across studies). This model may be estimated using standard statistical software packages for random effects models or multilevel models, such as SAS, Stata, and MLwiN [7, 24, 25].

Statistical models for network meta-analysis become more complex, when some of the included studies have more than 2 treatment arms. This is because the differences in treatment effects within a study with multiple arms are not independent. Currently, a Bayesian approach to network meta-analysis developed by researchers in the Bristol and Leicester Universities is the most popular approach in the literature and used for the analysis of our example data in the next section [26, 27]. The differences between non-Bayesian and Bayesian approaches are mainly computational (i.e., the statistical algorithms for obtaining the results) rather than conceptual (i.e., the basic statistical models are the same).

### 2.4. Bayesian Network Meta-Analyses

Bayesian network meta-analysis, also known as mixed treatments comparison, uses a Bayesian statistical framework for a synthesis of direct and indirect comparisons of different treatments [8–12, 14, 16]. In the Bayesian paradigm, prior beliefs about parameters in the models are specified and factored into the estimation. For example, the mean treatment effect may be specified as 0.5 mm and with a standard error of 2 mm. However, in most scenarios, a noninformative prior is usually specified, for example, the mean effect is zero with a extremely large standard error. Posterior distributions of model parameters are then derived from the prior information *and* observed data [28]. In the Bayesian network meta-analysis, noninformative prior is usually used to minimise the impact of prior information on final results, as evidence for supporting a specific treatment effect is generally lacking. A flat prior distribution such as a uniform distribution or a normal distribution with a large variance can be specified for a study on the difference in the therapeutic effects between two treatments; consequently, the posterior distributions were almost entirely derived from the observed data. For example, we can specify that the prior distribution of log hazard ratio in implant survival between two treatments for peri-implantitis follows a normal distribution with zero mean and variance of 10,000. Suppose the observed variance of the difference between periodontal treatments is usually around 3 and 5, within which our prior distribution will consequently look flat and is therefore noninformative. As the probability distribution within the range of possible values is the same, the posterior distribution for the difference in log hazard ratio is mainly determined by the observed data.

In Bayesian analysis, posterior distributions are usually quite complex, so software packages use simulations-based algorithms known as Markov Chain Monte Carlo method to derive the approximate posterior probability distributions. Markov Chain Monte Carlo method starts with a set of initial values and then runs an iteration process to obtain the approximate posterior distribution. Samples are then taken from these posterior distributions for calculating each parameter in the model. Because Bayesian approach is a simulations-based methodology, it also has several other advantages. For instance, it can estimate predicted treatment effects based on the observed data, and it can provide ranking for treatments, which is especially useful when the differences between treatments are small. The statistical software for Bayesian approach is also more flexible than standard software in adapting to each unique situation where, for example, some studies have more than two treatment arms and where studies have different designs, such as parallel-group and split-mouth design.

The downside to Bayesian approach is that the statistical theory and estimation method for Bayesian network meta-analysis are mathematically advanced, and the software for Bayesian analysis, such as WinBUGS, has a steep learning curve. The website of the department of community-based medicine in Bristol University (https://www.bris.ac.uk/cobm/research/mpes/mtc.html/) contains many WinBUGS code examples for network meta-analysis, and researchers can modify those codes for their analysis.

## 3. Network Meta-Analysis in Practice

### 3.1. Example Data

In this section, we used an example to illustrate how to undertake a network meta-analysis. We recently conducted a systematic review for the effectiveness of guided tissue regeneration (GTR), enamel matrix derivatives (EMD), and their combination therapies on the treatment of periodontal infrabony lesions [16]. In this tutorial, we used a subgroup of RCTs that compared GTR with nonresorbable or resorbable membranes to flap operation and that compared GTR with different types of membranes. The literature search strategy and quality assessment of included studies can be found in our previous publication. The outcome variable for our illustration is change in clinical attachment level (CAL), and we found 18 RCTs that compared at least two of the three treatments (flap operation, GTR with nonresorbable membranes, and GTR with resorbable membranes) [29–46]. One study [46] was then excluded, because it did not report the standard errors for the treatment effects for different GTR treatment groups. One study [35] had two GTR treatment groups with nonresorbable membranes, and results from the two groups were combined into one. Of the 17 studies, only one study [36] compared all three treatment groups.  Table 1 provided a summary for the 17 studies included in the final network meta-analysis.

### 3.2. Traditional Pairwise Comparisons

**Figure 2:**
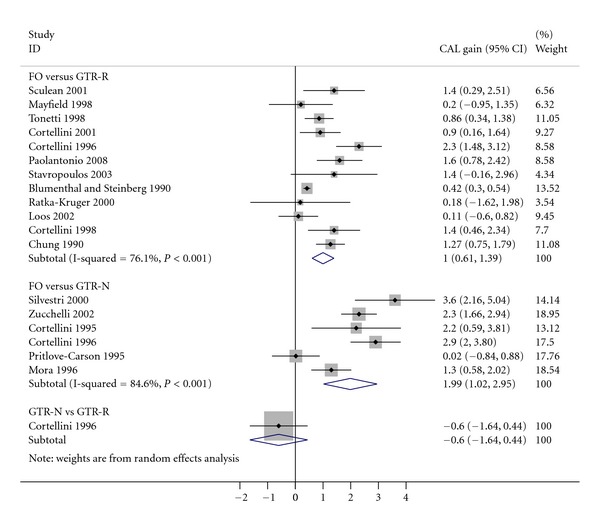
Forest plot for the three pairwise meta-analyses: flap operation (FO) versus GTR-N, FO versus GTR-R, and GTR-N versus GTR-R.

**Figure 3:**
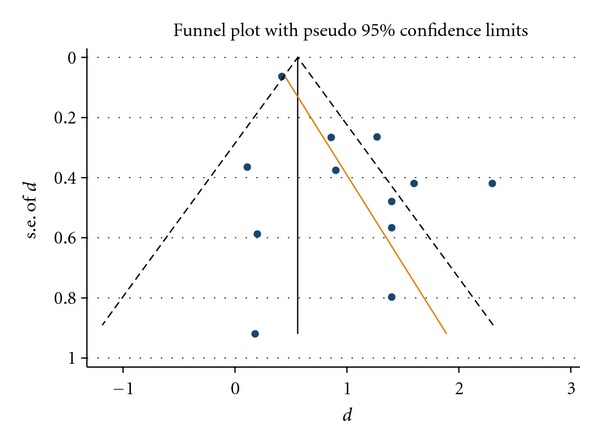
Funnel plot for the comparison between GTR-R and flap operation. The red line is fitted line from the Egger's test, indicating a small study bias as studies with small sample sizes tended to show greater treatment benefit for GTR-R.

Traditional pairwise comparisons only use the direct evidence, and they should be undertaken in order to evaluate the consistency in direct and indirect comparisons. We used statistical software Stata (Version 12, StataCorp, College Station, TX, USA) for the analysis, and results were shown in Figure 2. Six studies compared GTR with nonresorbable membrane (GTR-N) to flap operation, and results from random effects meta-analysis showed GTR-N achieved 1.99 mm (95% confidence intervals (CI): 1.02 to 2.95) greater CAL gain than flap operation. Twelve studies compared GTR with resorbable membranes (GTR-R) to flap operation, and results from random effects meta-analysis showed GTR-R achieved 1 mm (95% confidence intervals (CI): 0.61 to 1.39) greater CAL gain than flap operation. Both meta-analyses showed substantial heterogeneity: the Cochrane-Q test is statistically significant (*P* < 0.001), and the I-squared was 76% and 85%, respectively. Funnel plot and Egger test suggested there might be small study bias in the comparison between GTR-R and flap operation (Figure 3). Only one study compares the two GTR treatments, and its results showed that GTR-N achieved 0.6 mm (95%CI: −0.44 to 1.64) greater CAL gain than GTR-R.

### 3.3. Bayesian Network Meta-Analysis

**Figure 4:**
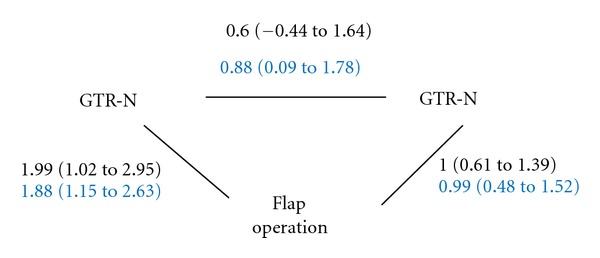
Diagram for the network meta-analysis. The width of lines is proportional to the number of studies included in the pairwise comparisons. The estimates for the differences in treatment effects from traditional meta-analysis were in black, whilst those from the Bayesian network meta-analysis were in blue.

Bayesian network meta-analysis was then undertaken using the software WinBUGS (MRC Biostatistics Unit, Cambridge, England). WinBUGS codes used in our analysis were a modification of codes available on the website of the department of community-based medicine in Bristol University to accommodate continuous outcomes and studies with split-mouth design. Noninformative priors were used throughout the analyses. Markov Chain Monte Carlo method with 50,000 burn-in and further 50,000 simulations and with three chains of different initial values (i.e., 150,000 simulations in total) was used to obtain medians and 95% credible intervals (i.e., the 2.5 and 97.5 percentiles of simulation results), which may be interpreted as the likely range of the estimated parameter by excluding the extreme values. Results from the Bayesian network meta-analysis using the 17 studies were similar to those from the traditional pairwise comparison: GTR-N and GTR-R achieved 1.88 mm (95% credible intervals (CrI): 1.15 to 2.63) and 0.99 mm (95% CrI: 0.48 to 1.52) greater CAL gain than flap operation, respectively; GTR-N achieved 0.88 mm (95% CrI: 0.09 to 1.78) greater CAL gain than GTR-R.  Figure 4 provided comparisons between results from the Bayesian and traditional meta-analysis.

### 3.4. Evaluating the Inconsistency between Direct and Indirect Evidence

Figure 4 showed that the three treatments in our network formed a loop, and, for every loop, it was possible to evaluate the consistency between direct and indirect evidence. For example, results from the single trial comparing GTR-N to GTR-R are direct comparison, whilst results from the traditional pairwise meta-analysis of the 6 trials comparing GTR-N to flap operation and those from the 12 trials comparing GTR-R to flap operation provide indirect comparisons between GTR-N and GTR-R by using flap operation as the reference treatment [47]. We denote the difference in CAL gain for comparing GTR-N to flap operation as *d*_*N*-*F*_, the difference in CAL gain for comparing GTR-R to flap operation as *d*_*R*-*F*_, and the difference in CAL gain for comparing GTR-N to GTR-R as *d*_*N*-*R*_. The indirect comparison between GTR-N and GTR-R is therefore *d*_*N*-*F*_ − *d*_*R*-*F*_ = 1.99 − 1 = 0.99 = *d*_*N*-*R*_^Ind^. The inconsistency is the difference between *d*_*N*-*R*_ and *d*_*N*-*R*_^Ind^: *δ* = *d*_*N*-*R*_ − *d*_*N*-*R*_^Ind^ = 0.99 − 0.6 = 0.39. A simple statistical test for evaluating the inconsistency is [47]:

(3) $$\begin{array}{c} z=\frac{\delta }{\sqrt{\sigma_{\delta }^{2}}}, \end{array}$$

where *z* is the ratio of *δ* over its standard error *σ*_*s*_ and follows a normal distribution. The variance *σ*_*δ*_^2^ can be estimated by *σ*_*δ*_^2^ = *σ*_*d*_*N*-*F*__^2^ + *σ*_*d*_*R*-*F*__^2^ + *σ*_*d*_*N*-*R*__^2^, where *σ*_*d*_*N*-*F*__^2^, *σ*_*d*_*R*-*F*__^2^, and *σ*_*d*_*N*-*R*__^2^ are variances of *d*_*N*-*F*_, *d*_*R*-*F*_, and *d*_*N*-*R*_, respectively. Using the results from Figure 2

(4) σδ2σdN-F2+σdR-F2+σdN-R2=(0.493)2+(0.201)2+(0.532)2=0.566,z=δσδ2=0.390.566=0.390.752=0.52.

The *P* value for *z* = 0.52 was 0.603, indicating that the inconsistency in direct and direct evidence was not significant. 

Note that, for any loop, results of testing inconsistency remain the same, irrespective of the reference group chosen from the loop.

### 3.5. Ranking for the Treatments

**Figure 5:**
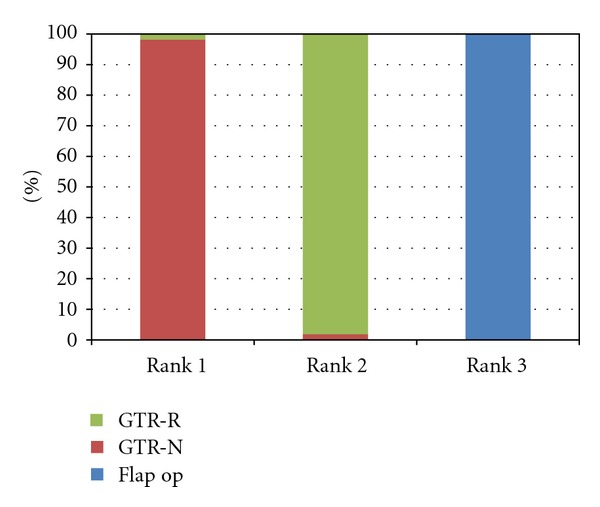
Treatment rankings. The bar chart showed the probability of each treatment for being the best, the second best, and the third in terms of CAL gain.

Because Markov Chain Monte Carlo method for estimation used by Bayesian analysis is a simulation-based approach, we can calculate the rank for each treatment according its performance in each simulation [48]. In 98.3% of simulations, GTR-N ranked the best amongst the three treatments, and, in 1.7% of simulations, GTR-R ranked the best; this is consistent with results for point estimates and their credible intervals. Information about Ranking is especially useful when the number of treatments in the network meta-analysis is large and the differences in their treatment effects are small.  Figure 5 shows the treatment rankings for all three treatments.

## 4. Practical Issues in Conducting a Network Meta-Analysis

As shown in our example, the main difference between a network meta-analysis and multiple pairwise meta-analyses for comparisons of multiple treatments is that the former use both direct and indirect evidence whilst the latter only use the direct evidence. All meta-analyses require comprehensive literature search, careful evaluation of available studies, and attentions to potential biases and heterogeneity. In this section, we discussed a few practical issues that may arise in conducting a network meta-analysis in dental research and provided some recommendations on how to deal with those issues.

### 4.1. Inclusion Criteria for Relevant Studies Need to Be Clearly Explained

Decisions on which studies should be included in the network meta-analysis are not always straightforward when undertaking network meta-analysis, because indirect evidence can be very broad [49, 50]. In our example, we included RCTs that compared at least two of the three treatments, GTR-N, GTR-R, and flap operation with the latter as the reference group. However, in terms of indirect comparisons, studies that contained one of even none of the three treatments could also be included. For example, studies that compared GTR-N to enamel matrix derivatives (EMD) or EMD to flap operation provided indirect evidence for comparing GTR-N with flap operation. Consequently, almost all RCTs on the treatment of periodontal infrabony lesions might be included in the network meta-analysis, and a practical decision has to be made for the inclusion criteria. This is why we decided that only studies that compared at least two of the three treatments were included. If the definition for relevant studies is too broad, the number of included studies will be large and results from network meta-analysis may be difficult to interpret due to the excessive heterogeneity and inconsistency in the evidence. The definition for relevant studies needs to be based on clinical knowledge; for instance, treatments or drugs that are either no longer used or available may be excluded. However, for a group of treatments within a network meta-analysis, the literature search should be comprehensive so that all eligible studies are included.

### 4.2. Data from Trials with Multiple Arms Should Be Appropriately Analysed

When there are more than two treatments in a trial, the study-specific treatment effects are unlikely to be independent; for example, suppose a trial has three treatment arms, GTR-N, GTR-R, and flap operation, and they are carried out by experienced periodontists. Consequently, treatment effects of both GTR-N and GTR-R compared to flap operation are likely to be greater than those in a trial where treatments are carried out by a periodontist in training. Special care has to be taken to avoid using the same control group more than once in estimating the differences in treatment effects between the test groups and the control group [25]. In our example, one study has three treatment arms, and it was assumed that the correlation between the two treatment effects differences (i.e., flap operation versus GTR-N, and flap operation versus GTR-R) was 0.5 following the suggestion by Lu and Ades [27]. The impact on the network meta-analysis of disregarding the correlations among treatment effects depends on the number of studies with more than two treatment arms and the strength of those correlations.

### 4.3. Possible Reasons for Any Observed Discrepancies between Direct and Indirect Evidence Should Be Investigated

In our example, there is no significant inconsistency in direct and indirect evidence, and, consequently, the discrepancy between results from traditional pairwise and Bayesian network meta-analyses is small. If the inconsistency test is significant, the discrepancy is likely to be large, as results from the Bayesian analysis are a combination of direct and indirect evidence. Substantial discrepancies usually indicate the heterogeneity, and similarity assumptions as discussed in Section 2.1 may be questionable, and researchers need to investigate the sources of heterogeneity. For instance, suppose treatment effects are related to baseline disease severity; if patients in A versus placebo trials have more severe baseline disease than those in B versus placebo trials, indirect comparisons will show that A seems to have greater treatment effects than B, even if there is no difference between A and B. In other scenarios, direct evidence comparing A with B may have a dubious quality and suffer a greater bias; consequently, indirect evidence becomes more reliable [51]. Heterogeneity is an issue that should be investigated in any meta-analysis (including both pairwise and network meta-analyses), and, when there is inconsistency in direct and indirect evidence, characteristics of studies involved in comparisons should be carefully evaluated; if feasible, a meta-regression or subgroup analysis should be undertaken to explore the sources of heterogeneity [21–23].

### 4.4. Missing Information May Be Imputed Using Simple Methods

The most common missing information encountered in conducting a meta-analysis in dental research is the standard errors for the mean treatment effects which are usually used as the weights for combining the treatment effects reported by different studies, when the outcomes are continuous variables such as CAL. For binary outcomes, such as success or survival, treatment effects and its confidence intervals are derived from the numbers of patients in different categories, for example, the numbers of patients with or without success in different treatment groups. When the outcome is a continuous variable, we need the mean and its standard error for meta-analysis. The most common scenario is that a study reported the mean treatment effects and their standard errors without reporting the difference in treatment effects and its confidence intervals.  Table 2 shows results from a hypothetic study with two treatments to illustrate how to impute the missing standard errors.

The change in the outcome CAL for the test group is 4.3 mm with standard deviation 1.3 mm, and the change in the outcome CAL for the control group is 1.9 mm with standard deviation 1.1 mm. Suppose this study uses a parallel-group design and has 10 patients in each group; the mean difference in treatment effects between the test and control groups is 4.3 − 1.9 = 2.4, and its standard error (*σ*_*D*_) can be obtained by using the following formula [52]:

(5) $$\begin{array}{c} \sigma_{D}=S\sqrt{\left(\frac{1}{n_{T}}+\frac{1}{n_{C}}\right)}, \end{array}$$

where

(6) $$\begin{array}{c} S=\sqrt{\left[\frac{\left(n_{T}-1\right)s_{T}^{2}+\left(n_{C}-1\right)s_{C}^{2}}{\left(n_{T}+n_{C}-2\right)}\right]}, \end{array}$$

*n*_*T*_ and *n*_*C*_ are the number of patients in the treatment and control groups, respectively, and *S*_*T*_ and *S*_*C*_ are the standard deviations of treatment effects for the treatment and control groups, respectively. Using the information in Table 2, we can obtain the pooled standard deviation *S*:

(7) $$\begin{array}{c} S=\sqrt{\left[\frac{9{\left(1.3\right)}^{2}+9{\left(1.1\right)}^{2}}{18}\right]}=\sqrt{\frac{1.69+1.21}{2}}=1.204,\\ \sigma_{D}=1.204\sqrt{\left(\frac{1}{10}+\frac{1}{10}\right)}=0.54. \end{array}$$

The *t*-value for the mean difference in treatment effects between the test and control groups is therefore 2.4/0.54 = 4.44, and the *P* value is 0.0003.

In a study with parallel-group design, the two treatments effects are supposed to be independent, but, in a study with split-mouth design (e.g., two teeth of the same patient are randomly assigned to the test and control groups), the treatment effects are likely to be correlated [53] and (6) is no longer suitable for obtaining the pooled standard deviation *S*. Instead, we use the following formula to obtain *S*:

(8) $$\begin{array}{c} \sigma_{D}=\frac{S}{\sqrt{n}}=\sqrt{\frac{s_{T}^{2}+s_{C}^{2}-2rs_{T}s_{C}}{n},} \end{array}$$

where *n* is the sample size and *r* is the correlation coefficient between the two treatment effects. The only missing information is *r*, and we have to make an informed guess about it. Lesaffre et al. [53] suggested using *r* = 0.25 for split-mouth study. Using Table 2 and *n* = 10,

(9) $$\begin{array}{c} \sigma_{D}=\frac{S}{\sqrt{n}}=\sqrt{\frac{\left(1.69+1.21-0.5*1.3*1.1\right)}{10}}=0.47. \end{array}$$

Sometimes, a study only reported the means and standard deviations for the baseline and follow-up measurements for each group, and we need to calculate the means and standard deviations for change in the outcome for both groups first before we can apply either (6) or (8). Suppose *x*_1_ and *x*_2_ are the baseline and follow-up CAL for the test group, and *d* is the difference in CAL between *x*_1_ and *x*_2_. The mean of *d* is the difference in the means of *x*_1_ and *x*_2_, and the variance of *d*(*s*_*D*_^2^) can be obtained using the following formula: *s*_*D*_^2^ = *s*_1_^2^ + *s*_2_^2^ − 2*rs*_1_ *s*_2_, where *s*_1_ and *s*_2_ are the standard deviations for *x*_1_ and *x*_2_, respectively, and *r* is the correlation coefficient between *x*_1_ and *x*_2_. Again, we need to make an informed guess about *r*, and *r* = 0.5 seems to be a reasonable one [54].

Imputing missing values can increase the number of included studies in a meta-analysis, and this is especially useful for a network meta-analysis when the number of treatments in comparisons is large but the number of studies is relatively few.

## 5. Conclusion

Network meta-analysis is an extension of pairwise comparisons of treatments to comparisons of all available treatments by incorporating both direct and indirect evidence. This new methodology has become widely adopted by meta-analysts in medical research and has been proven to be a very useful tool for evidence synthesis. The use of more sophisticated statistical approaches such as network meta-analysis can improve the efficiency in comparative effectiveness research and in the quality of decisions making. This tutorial aims to bring this new methodology to the attentions of dental researchers and to facilitate its adoption but also highlight several important issues in conducting and interpreting a network meta-analysis. Further information and technical details can be found in [3, 4, 25–27]. The statistical theory behind network meta-analysis is nevertheless complex, so we strongly encourage close collaboration between dental researchers and experienced statisticians when planning and conducting a network meta-analysis.

## Figures and Tables

**Table 1:** Summary of studies included in the network meta-analysis for CAL gain. SE: standard error; FO: flap operation; GTR-N: guided tissue regeneration with nonresorbable membranes; GTR-R: guided tissue regeneration with resorbable membranes.

Study	Mean	SE	Treatment	Study design
Sculean et al. 2001 [29]	1.70	0.40	FO	parallel-group
Sculean et al. 2001 [29]	3.10	0.40	GTR-R	parallel-group
Silvestri et al. 2000 [30]	1.20	0.33	FO	parallel-group
Silvestri et al. 2000 [30]	4.80	0.66	GTR-N	parallel-group
Zucchelli et al. 2002 [31]	2.60	0.15	FO	parallel-group
Zucchelli et al. 2002 [31]	4.90	0.29	GTR-N	parallel-group
Mayfield et al. 1998 [32]	1.30	0.40	FO	parallel-group
Mayfield et al. 1998 [32]	1.50	0.42	GTR-R	parallel-group
Tonetti et al. 1998 [33]	2.18	0.18	FO	parallel-group
Tonetti et al. 1998 [33]	3.04	0.20	GTR-R	parallel-group
Cortellini et al. 2001 [34]	2.60	0.24	FO	parallel-group
Cortellini et al. 2001 [34]	3.50	0.28	GTR-R	parallel-group
Cortellini et al. 1995 [35]	2.50	0.46	FO	parallel-group
Cortellini et al. 1995 [35]	4.70	0.53	GTR-N	parallel-group
Cortellini et al. 1996 [36]	2.30	0.23	FO	parallel-group
Cortellini et al. 1996 [36]	5.20	0.40	GTR-N	parallel-group
Cortellini et al. 1996 [36]	4.6	0.35	GTR-R	parallel-group
Paolantonio et al. 2008 [37]	1.50	0.25	FO	parallel-group
Paolantonio et al. 2008 [37]	3.10	0.34	GTR-R	parallel-group
Stavropoulos et al. 2003 [38]	1.50	0.58	FO	parallel-group
Stavropoulos et al. 2003 [38]	2.90	0.54	GTR-R	parallel-group
Blumenthal and Steinberg 1990 [39]	0.75	0.06	FO	split-mouth
Blumenthal and Steinberg 1990 [39]	1.17	0.03	GTR-R	split-mouth
Pritlove-Carson et al. 1995 [40]	1.73	0.36	FO	split-mouth
Pritlove-Carson et al. 1995 [40]	1.78	0.45	GTR-N	split-mouth
Ratka-Kr$\ddot{\text{u}}$ger et al. 2000 [41]	4.00	0.77	FO	split-mouth
Ratka-Kr$\ddot{\text{u}}$ger et al. 2000 [41]	4.18	0.64	GTR-R	split-mouth
Loos et al. 2002 [42]	1.29	0.31	FO	split-mouth
Loos et al. 2002 [42]	1.40	0.28	GTR-R	split-mouth
Cortellini et al. 1998 [43]	1.60	0.38	FO	split-mouth
Cortellini et al. 1998 [43]	3.00	0.35	GTR-R	split-mouth
Chung et al. 1990 [44]	−0.71	0.29	FO	split-mouth
Chung et al. 1990 [44]	0.56	0.18	GTR-R	split-mouth
Mora et al. 1996 [45]	2.55	0.32	FO	split-mouth
Mora et al. 1996 [45]	3.85	0.28	GTR-N	split-mouth

**Table 2:** Results from a hypothetic study with two treatment groups. The outcome is the mean clinical attachment level (CAL) and the standard deviation in brackets.

CAL	Baseline	Followup at 12 months	Change
Test	10.5 (1.9)	6.2 (1.5)	4.3 (1.3)
Control	10.3 (1.8)	8.4 (1.6)	1.9 (1.1)
